# Ultrafast Coherent THz Lattice Dynamics Coupled to Spins in the van der Waals Antiferromagnet FePS_3_

**DOI:** 10.1002/adma.202208355

**Published:** 2022-12-21

**Authors:** Fabian Mertens, David Mönkebüscher, Umut Parlak, Carla Boix‐Constant, Samuel Mañas‐Valero, Margherita Matzer, Rajdeep Adhikari, Alberta Bonanni, Eugenio Coronado, Alexandra M. Kalashnikova, Davide Bossini, Mirko Cinchetti

**Affiliations:** ^1^ Department of Physics TU Dortmund University Otto‐Hahn Straße 4 44227 Dortmund Germany; ^2^ Instituto de Ciencia Molecular (ICMol) Universidad de Valencia Catedrático José Beltrán 2 Paterna 46890 Spain; ^3^ Institute of Semiconductor and Solid State Physics Johannes Kepler University Linz Altenbergerstr. 69 Linz 4040 Austria; ^4^ Ioffe Institue St. Petersburg 194021 Russia; ^5^ Department of Physics and Center for Applied Photonics University of Konstanz D‐78457 Konstanz Germany

**Keywords:** 2D materials, antiferromagnets, magnon, phonon, spintronics, ultrafast pump‐probe spectroscopy, van der Waals semiconductors

## Abstract

Coherent THz optical lattice and hybridized phonon–magnon modes are triggered by femtosecond laser pulses in the antiferromagnetic van der Waals semiconductor FePS_3_. The laser‐driven lattice and spin dynamics are investigated in a bulk crystal as well as in a 380 nm‐thick exfoliated flake as a function of the excitation photon energy, sample temperature and applied magnetic field. The pump‐probe magneto‐optical measurements reveal that the amplitude of a coherent phonon mode oscillating at 3.2 THz decreases as the sample is heated up to the Néel temperature. This signal eventually vanishes as the phase transition to the paramagnetic phase occurs, thus revealing its connection to the long‐range magnetic order. In the presence of an external magnetic field, the optically triggered 3.2 THz phonon hybridizes with a magnon mode, which is utilized to excite the hybridized phonon–magnon mode optically. These findings open a pathway toward the optical control of coherent THz photo–magnonic dynamics in a van der Waals antiferromagnet, which can be scaled down to the 2D limit.

## Graphical abstract

A step toward the optical generation and control of coherent THz spin and lattice dynamics in 2D antiferromagnets. A coherent hybrid phonon–magnon mode is triggered in the 2D antiferromagnet FePS_3_ by pumping below the band gap in the presence of an external magnetic field.

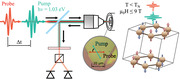

## 1 Introduction

The synthesis of few‐atomic‐layers‐thin materials^[^ ^1^, ^2^, ^3^ ^]^ has ignited the spark of a massive research effort aiming at manipulating their macroscopic properties. More recently, 2D magnetically ordered materials have been produced as well.^[^ ^4^, ^5^, ^6^, ^7^ ^]^ The long‐range magnetic order in these compounds appears to be highly susceptible to lattice distortions, rooted in the role of the magnetic anisotropy in the stabilization of the long‐range order in 2D magnets.^[^ ^8^ ^]^ The ultrafast generation of phonons, via a variety of mechanisms, has been proved to be a powerful tool for driving and controlling spin dynamics in bulk magnets at fundamental timescales.^[^ ^9^, ^10^, ^11^, ^12^, ^13^, ^14^ ^]^ This route is also viable for crystals of van der Waals 2D materials, as recently demonstrated by the presence of dynamic spin‐lattice coupling in a ferromagnetic CrI_3_ crystal.^[^ ^15^ ^]^ In this context, 2D antiferromagnets offer several fundamental advantages in spintronic perspective in comparison with ferromagnets. Their main benefits lie in their much more stable ground state as well as in the magnetic resonance frequencies in the THz range, which are orders of magnitude higher than in ferromagnets. Crucially, the coupling of antiferromagnetic magnons to phonons is in the energy range of optical phonons, which led to recent reports of hybridized magnon‐phonon quasiparticles in 2D antiferromagnetic materials.^[^ ^16^, ^17^, ^18^, ^19^, ^20^ ^]^ Optically driven collective lattice modes carry therefore potential for the optical control of the long‐range magnetic order in 2D antiferromagnets, based on the well‐established possibility to drive such modes fully coherently even with a photon energy far from their eigenfrequency^[^ ^21^, ^22^ ^]^ and on their strong coupling to magnons. In this context, transition metal trichalcogenophosphates (MPX_3_, with M = Ni, Fe, Mn, … and X = S, Se) represent an interesting class of van der Waals antiferromagnets.^[^ ^23^, ^24^, ^25^, ^26^ ^]^ While the optical generation of magnons has been reported in a free‐standing NiPS_3_ bulk single‐crystal^[^ ^27^ ^]^ , this material lacks from scalability down to the 2D limit. In fact, it has been experimentally demonstrated that a single atomic layer of NiPS_3_ is not magnetically ordered^[^ ^26^ ^]^ differently from MnPS_3_^[^ ^28^ ^]^ and FePS_3_.^[^ ^25^ ^]^

## 2 Results and Discussion

In this work, we therefore select FePS_3_ as a van der Waals antiferromagnet scalable to the 2D limit and investigate first a free‐standing bulk‐crystal, and then an exfoliated flake with a lateral size of ≈50 µm and thickness of ≈380 nm deposited on a SiO_2_/Si substrate (see Supporting Information for details on sample growth and characterization). The flake represents a structure that can be scaled down to the 2D limit – one monolayer – without losing the antiferromagnetic order^[^ ^25^, ^29^ ^]^ and which is more stable than other 2D magnets (as CrI_3_).^[^ ^30^ ^]^ We demonstrate the laser‐induced excitation of two coherent lattice modes in the bulk crystal with frequencies of 3.2 THz and 4.8 THz. The 3.2 THz mode is observed also in the flake sample and, crucially, it is intimately coupled to the long‐range magnetic order, since its amplitude vanishes above the Néel temperature. Moreover, this mode hybridizes with a zone‐center magnon mode under the application of an external field^[^ ^18^, ^19^, ^20^ ^]^ that we exploit here to optically excite the hybrid phonon–magnon mode.

**Figure 1:**
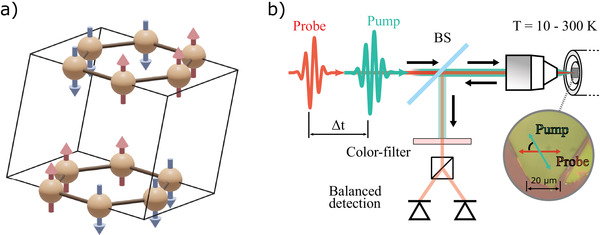
a) Fe‐ions within the crystallographic unit cell of FePS_3_. b) Schematic view of the experimental setup for the time‐resolved investigations of flake samples. The pump (0.83–1.08 eV) and probe (1.45 eV) beams are collinear and focused on the 2D flake with a microscope objective.

The crystal and magnetic structure of FePS_3_ is shown in **Figure** **1**a. Below the Néel temperature (*T*_N_ ≈118 K^[^ ^25^, ^31^ ^]^), the magnetic moments of the Fe‐ions are oriented out‐of‐plane and form a zigzag pattern within the layers, which build up to a monoclinic crystal structure with *C*2*/m* spacegroup.^[^ ^32^ ^]^ The band‐gap energy of FePS_3_ is reported at ≈1.5 eV at room temperature ^[^ ^33^, ^34^ ^]^ while a d−d transition ≈1.1 eV appears in the absorption spectrum, as reported in the literature.^[^ ^35^, ^36^ ^]^ To detect the optically‐induced phonon and magnon dynamics, we employ pump‐probe optical spectroscopy and measure the photo‐induced rotation of the probe polarization. The literature abundantly demonstrates that this experimental scheme is able to monitor the lattice dynamics.^[^ ^21^ ^]^ In magnetic materials displaying quadratic magneto‐optical effects, our experimental geometry allows to track both the longitudinal and transverse dynamics of the Néel vector.^[^ ^37^, ^38^, ^39^, ^40^ ^]^ This is the case in FePS_3_, where quadratic effects are indeed enabled by the breaking of rotational symmetry induced by the zigzag pattern of the magnetic moments.^[^ ^41^, ^42^ ^]^

The experimental setup for pump‐probe optical spectroscopy consists of an amplified laser system with a repetition rate of 200 kHz. The main output of the laser (20 W average output power) is split into two beams (13 W and 7 W) seeding two optical parametric amplifiers (OPA), which generate laser beams with photon energy in the 0.5–3.5 eV range. The outputs of the OPAs, whose photon energies can be tuned independently of each other, are employed as pump and probe beams, as described elsewhere.^[^ ^43^ ^]^ The sample is mounted on a piezo‐driven three‐axis‐stage inside a cryostat. For the measurements on the bulk crystal, the cryostat is placed inside a superconducting magnet, able to generate a magnetic field as intense as 9 T, while the pump beam is focused by a spherical mirror, keeping the focus position practically independent of the choice of the photon‐energy. To investigate the flake sample, a collinear pump‐probe scheme, as shown in Figure 1b, is employed. Both pump and probe beams are focused on the flake with an objective down to a spot of 1.5 µm diameter, as estimated by knife‐edge measurements. This configuration allows to fully focus both beams into spots on different parts of the flake, so that homogeneous regions can be addressed in samples with lateral size larger than 5 µm.^[^ ^44^ ^]^

**Figure 2:**
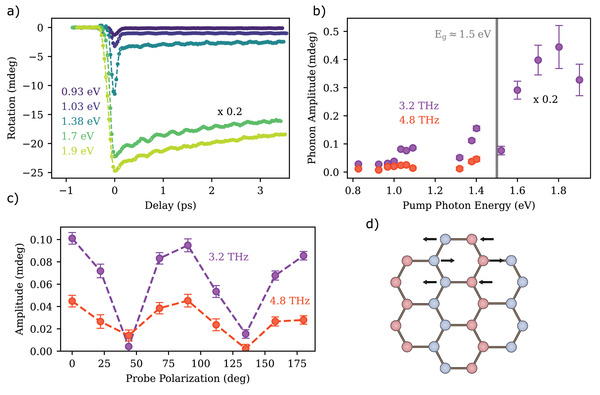
a) Transient rotation of the polarization measured at 10 K for different pump photon‐energies. The pump and probe beam were linearly polarized 45° away from each other. The pump fluence is kept constant to 2 mJ cm^−^^2^ for pump photon‐energy below the band gap and to 1 mJ cm^−^^2^ for pump photon‐energy above the band gap. b) Amplitude of the 3.2 THz (purple) and the 4.8 THz (orange) phonon modes as a function of the pump photon‐energy. c) Probe polarization dependence of the two observed phonon modes. d) Schematic representation of the Fe‐ions lattice in FePS_3_, forming a magnetic zig‐zag pattern. The arrows indicate exemplarily the motion of the ions in a Raman‐active phonon mode.

First, we now focus on the optically excited dynamics in the bulk crystal. For these experiments the probe photon‐energy was kept constant at 1.45 eV, which is just below the band‐gap energy of 1.5 eV^[^ ^33^, ^34^ ^]^ while the pump photon energy was tuned from below to above the band gap of FePS_3_ in the 0.83–1.9 eV range. **Figure** **2**a reports the transient rotation measured for different pump photon‐energies with the sample temperature set to 10 K. The data display both a coherent and incoherent contribution, which are strongly dependent on the excitation photon‐energy. We first analyze the coherent signal by applying a Fourier transform as explained in more details in the Supporting Information. The results reveal a superposition of two coherent oscillations with frequency of 3.2 THz and 4.8 THz. Both values match the eigenfrequencies of Raman‐active optical phonons reported in the literature.^[^ ^25^, ^29^, ^31^, ^45^ ^]^ Therefore, the coherent oscillations observed in the rotational signal can be ascribed to changes in either the linear crystallographic birefringence or dichroism, induced by the phonon‐modulated magnetic zigzag pattern, as schematically shown for one mode in Figure 2d. This is also confirmed by the probe polarization dependence shown in Figure 2c, following the two‐fold symmetry of the crystal surface with the highest response at 45° relative to the symmetry axis (the dependence on the pump polarization is shown in the Supporting Information). We stress that the 3.2 THz phonon mode is also visible in the transient reflectivity (see Supporting Information). However, within our experiment, the rotation of polarization is more sensitive to the phonon mode. The phonon lifetime was determined by scanning longer delay times (see Supporting Information). We obtained (21 ± 2) ps for excitations below and (5 ± 1) ps for excitations above the band gap. We ascribe the decrease in the lifetime to the increased scattering probability with incoherent phonons and hot electrons generated for resonant pumping.

The data shown in Figure 2a are just a few selected traces of the entire set measured as a function of the pump photon‐energy. Summarizing all the measurements, we visualize the spectral dependence of the phonon amplitude in the bulk sample in Figure 2b. The data were obtained by fitting each data set to a function consisting of an incoherent background superimposed to harmonic oscillations (see Supporting Information). The extracted amplitude of the phonon modes increases in the presence of electronic transitions, i.e., in the region ≈1.1 eV (d–d‐ transitions) and then above 1.5 eV (the energy of the band gap), where we observe a steep slope related to the onset of the band‐gap. Considering this behavior, we argue that the phonon modes are excited via a displacive mechanism, where the excitation of electrons into unoccupied states alters the inter‐ionic potentials causing collective lattice movements to the minima of the new potential. Generally, the excitation process can also originate from impulsive stimulated Raman scattering. However, as the laser pulses are relatively long compared to the phonon period, the determination of the oscillation phase strongly depends on the identification of the zero‐delay position and is unreliable in this case.

**Figure 3:**
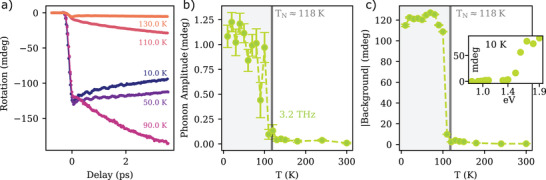
a) Transient rotation measured as a function of sample temperature with a pump photon energy of 1.9 eV. b) Amplitude of the 3.2 THz mode as a function of the sample temperature. c) Temperature dependence of the absolute value of the incoherent background. Inset: dependence of the incoherent background on the pump photon energy.

Seeking to experimentally establish a connection between the coherent phonon and the long‐range magnetic order, we measured the temperature dependence of the pump‐induced rotation of the probe polarization in the crystal. The time traces obtained by setting the pump photon energy to 1.9 eV are shown in **Figure** **3**a; they reveal a pronounced dependence of both the phonon amplitude and the background signal on the sample temperature. Figure 3b shows the temperature dependence of the amplitude of the 3.2 THz phonon mode, which vanishes above the Néel temperature. The phonon mode can thus be induced and detected only in the presence of the long‐range antiferromagnetic order. We note that this is fully consistent with the symmetry of the lattice mode, which is a zone‐folded mode. These lattice collective excitations appear in the dispersion of the material only in the antiferromagnetic phase, as the paramagnetic to antiferromagnetic phase transition takes place in concomitance to a doubling of the unit cell, and thus halving of the Brillouin zone.^[^ ^31^ ^]^

Figure 3c shows the absolute value of the incoherent background as a function of temperature. This component of the signal is only present in the magnetically ordered phase as well. Moreover, the inset of Figure 3c shows that the signal is much stronger in case of excitation with photon‐energy above the band‐gap. A visual inspection of the data in Figure 3a reveals that the characteristic time associated with the incoherent background increases as the Néel temperature is approached. This behavior is in agreement with a critical slow‐down of photo‐induced incoherent spin dynamics, that has been already reported in FePS_3_.^[^ ^41^ ^]^ Similar features have been observed in the rotation of the probe polarization, and interpreted as demagnetization of the antiferromagnetic sublattices, provided that quadratic magneto‐optical effects are allowed in the used experimental geometry.^[^ ^41^, ^46^, ^47^ ^]^ Summing up all these considerations, we thus ascribe the incoherent background to the demagnetization of the two Fe^2+^ sublattices triggered by photon absorption and dissipative processes.^[^ ^38^, ^48^ ^]^

**Figure 4:**
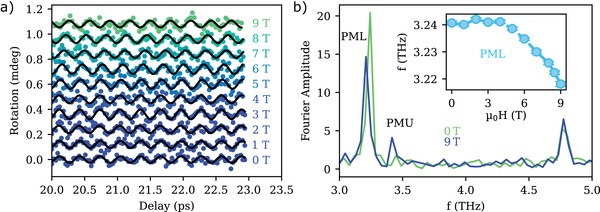
a) Magnetic field dependence of the rotation 20 ps after pump excitation at 1.03 eV. b) Fast Fourier‐transformation of the extended time‐traces with the absence of an external field (green) and at 9 T (blue). PML labels the peak of the lower branch of the phonon–magnon mode, which corresponds to the 3.2 THz phonon when no external field is applied. The peak labeled PMU is the upper branch phonon–magnon mode, appearing at 9 T. The other peak corresponds to the 4.8 THz phonon mode, which is unaffected by the external magnetic field. The inset shows the frequency shift of the PML mode caused by the phonon–magnon hybridization.

Let us now explore the hybridization of the 3.2 THz phonon to a zone‐center magnon by applying an external magnetic field up to 9 T to the crystal. **Figure** **4**a shows the transient rotation detected 20 ps after optical excitation as a function of the applied external field. Using this rotation signal at late delays it is possible to make even minor variations in the phonon frequency visible. In fact, for an applied field more intense than 5 T, a reduction in the oscillation frequency becomes indeed visible as an apparent shift in the phase, that can be appreciated by observing the interception of the curves with the *y*‐axis. To convert such phase shift Δφ into the corresponding change in frequency ω, we relate the two quantities using the formula Δφ = *t*(ω(0 T) − ω(μ_0_H)), with *t* = 21.4 ps and ω (0 T) = (3.2407±0.0006) THz, and obtain the data shown in the inset of Figure 4b, where it is clear that the frequency decreases with increasing magnetic field (more information on data analysis can be found in the Supporting Information). The numeric Fourier transformation of the data recorded at 0 and 9 T for delays between 0.1 and 24 ps is shown in Figure 4b and confirms this trend. We label this peak as PML, to distinguish it from a second peak present in the Fourier transform of the 9 T data at (3.42 ± 0.04) THz, that we label as PMU. These observations can be explained by considering that, in the presence of an external magnetic field, the phonon mode at 3.2 THz hybridizes with a magnon mode with an eigenfrequency of 3.6 THz at 0T^[^ ^18^, ^19^, ^25^, ^31^, ^45^ ^]^ giving rise to two phonon–magnon branches^[^ ^18^, ^19^, ^20^ ^]^: the phonon–magnon lower branch, PML, and the phonon–magnon upper branch, PMU. Since the pure magnon mode is not detectable in our experiments at 0 T, we deduce that it is the additional phononic component resulting from the hybridization that makes the PMU and PML modes detectable in our experiments. Crucially, the magnetic field dependence of the PML can be taken as experimental proof of the phonon–magnon hybridization and of the fact that we are indeed exciting the hybrid mode optically, by pumping FePS_3_ below the band gap. This is a very important step toward the generation and control of coherent THz spin and lattice dynamics in van der Waals antiferromagnets. For example, we propose that coherent control of amplitude and phase (as reported in reference^[^ ^38^ ^]^) could also be implemented on the hybridized mode.

**Figure 5:**
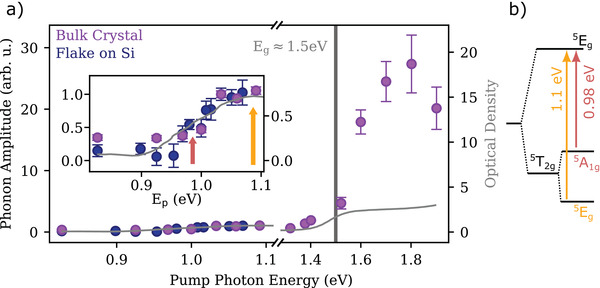
a) Amplitude of the 3.2 THz signal of the flake sample (blue) compared to the signal from the bulk crystal (purple). Both data set have been measured at *T* = 10 K and normalized to the value at 1.03 eV. The grey curve is the optical absorption of FePS_3_ measured in reference ^[^ ^35^ ^]^. b) Schematic of the d–d electronic transitions between the crystal field 3d‐state of the Fe^2+^ ions split by the octahedral and trigonal ligand fields.^[^ ^42^ ^]^

We now turn to the investigation of the flake sample, which should serve as a proof of principle for the scalability of our approach for flakes transferred on a substrate that can be produced as thin as a monolayer down to the 2D limit. In these experiments, the pump photon energy is kept below 1.1 eV, to avoid detrimental excitation of electrons in the conduction band of the silicon substrate. **Figure** **5**a shows the dependence of the 3.2 THz mode from the pump photon energy in the flake compared to the bulk crystal. Both samples show the same behavior, where the amplitude of the phonon follows the trend of the absorption spectrum (grey line). In the region ≈1.1 eV, optical absorption in FePS_3_ is dominated by d−d transitions of the Fe^2+^ ions, whose energy levels are split by the crystal field of the surrounding ligands. In particular, the two features at 0.98 eV and 1.1 eV can be ascribed to the optical transitions depicted in Figure 5b.^[^ ^49^ ^]^ The possibility of exciting optical phonons in this photon energy range is extremely important especially for the flake sample, as using photon energies below the band gap will prevent thermal damage of the low dimensional structures.

**Figure 6:**
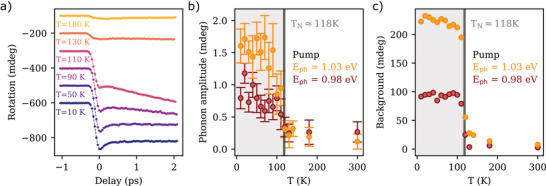
a) Pump‐induced rotation of the probe polarization detected at different values of temperature. The excitation photon energy was set to 1.03 eV. b) Temperature dependence of the 3.2 THz phonon amplitude for excitation photon‐energies of 1.03 and 0.98 eV. c) Incoherent background contribution as a function of temperature for excitation photon‐energies of 1.03 and 0.98 eV. The error bars lie within the markers.

Before concluding, we show in **Figure** **6**a the temperature dependence of transient rotation measured on the flake with a pump photon energy of 1.03 eV. We have measured a further data set for pump photon energy of 0.98 eV. Figure 6b,c shows respectively the amplitude of the 3.2 THz mode and of the incoherent background as a function of temperature. The results reveal the same trends discussed before in the bulk, where both the zone‐folded phonon as well as the incoherent background vanish above *T*_N_. We take this correspondence as a confirmation that our approach can be successfully applied also to flakes.

## 3 Conclusion

In conclusion, we have demonstrated THz coherent phonon–magnonic dynamics and incoherent spin dynamics in the antiferromagnetic phase of a bulk FePS_3_ crystal, driven by femtosecond laser pulses in a region of weak absorption. We have provided experimental evidence that our results can be scaled down to thinner flakes, by demonstrating the generation of the same coherent lattice mode in a 380 nm thin FePS_3_ flake deposited on a SiO_2_/Si substrate. Our results are extremely promising, especially in terms of recent advances in spintronic devices based on 2D materials and in their large‐scale co‐integration with conventional microelectronics materials.^[^ ^50^ ^]^ Here we highlight FePS_3_ as a promising 2D antiferromagnet where the coupling between phonon and magnons could be exploited to implement schemes for the optical control of magnetism at THz frequencies in miniaturized photo–magnonic devices, scalable down to the 2D limit.

## Conflict of Interest

The authors declare no conflict of interest.

## Supporting information

Supporting Information

## Data Availability

The data that support the findings of this study are available in the ZENODO database at https://doi.org/10.5281/zenodo.7383138.
