# Supplementary material for: Ultrafast Coherent THz Lattice Dynamics Coupled to Spins in the van der Waals Antiferromagnet FePS3
Source: Adv Mater. 2022 Dec 21;35(6):2208355. doi: 10.1002/adma.202208355 (PMC11475339; doi:10.1002/adma.202208355)
Supplement: Supplementary file 1 — Supporting Information [file ADMA-35-2208355-s001.pdf]

ADVANCED MATERIALS

Supporting Information

for *Adv. Mater.,* DOI: 10.1002/adma.202208355

Ultrafast Coherent THz Lattice Dynamics Coupled to Spins in the van der Waals Antiferromagnet $FePS_3$

*Fabian Mertens, David Mönkebüscher, Umut Parlak, Carla Boix-Constant, Samuel Mañas-Valero, Margherita Matzer, Rajdeep Adhikari, Alberta Bonanni, Eugenio Coronado, Alexandra M. Kalashnikova, Davide Bossini, and Mirko Cinchetti**

Ultrafast coherent THz lattice dynamics coupled to spins in the van der Waals antiferromagnet $FePS_3$

F. Mertens,[1] D. Mönkebüscher,[1] U. Parlak,[1] C. Boix-Constant,[2] S. Mañas-Valero,[2]

M. Matzer,[3] R. Adhikari,[3] A. Bonanni,[3] E. Coronado,[2] A. M. Kalashnikova,[4] D. Bossini,[5,1]

and M. Cinchetti[1]

[1)]*Department of Physics, TU Dortmund University, Otto-Hahn Straße 4, 44227 Dortmund, Germany*

[2)]*Instituto de Ciencia Molecular (ICMol) Universidad de Valencia. Catedrático José Beltrán 2 46890, Paterna, Spain*

[3)]*Institute of Semiconductor and Solid State Physics, Johannes Kepler University Linz, Altenbergerstr. 69, 4040 Linz, Austria*

[4)]*Ioffe Institue, 194021 St. Petersburg, Russia*

[5)]*Department of Physics and Center for Applied Photonics, University of Konstanz, 78464 Konstanz, Germany*

I. FLAKE THICKNESS

Figure S1(a) shows the atomic force microscopy (AFM) image of the flake sample that was used to calculate its thickness by measuring the height difference along the line scan shown in the figure. The line scan is taken across the edge between the flake and the substrate and shown in figure S1(b), together with the resulting flake thickness of 377 nm.

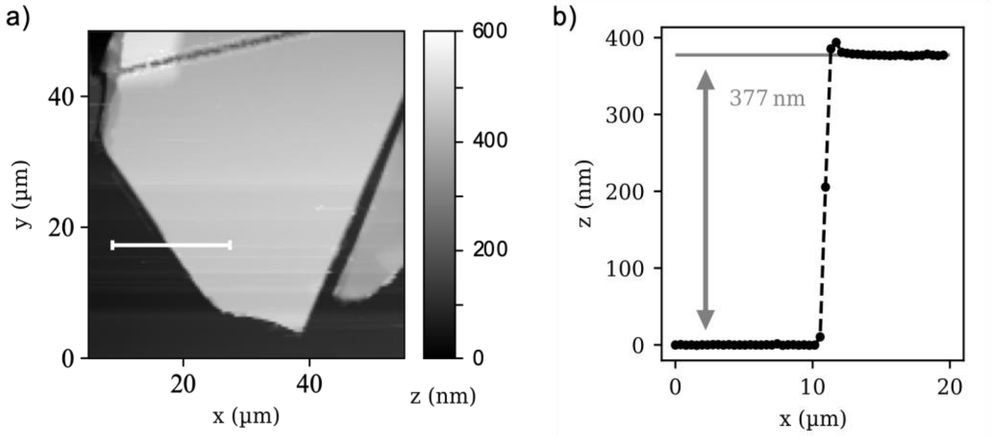

Figure S1. (a) AFM image of the investigated flake. (b) Height profile between the flake und substrate surface across the line scan indicated in (a).

II. DATA ANALYSIS

We describe here the data processing that was used to analyze the data reported in figures 2, 3, 5 and 6 from the main text, aimed at the quantitative evaluation of both the coherent and incoherent contributions to the detected rotation of the probe polarization. We fit the rotation of polarization signal at positive delays with a function that describes the coherent oscillations plus an estimation of the incoherent background $g(t)$. Depending on the excitation photon energy E_{ph} with respect to the band gap energy E_g, one of the two functions below is used to describe the coherent oscillations:

$$f_{Eph<Eg}(t) = P_1\, sin(\omega_1 t + \phi_1) + P_2 sin(\omega_2 t + \phi_2) + g(t) \quad (1)$$

$$f_{Eph>Eg}(t) = P_1\, sin(\omega_1 t + \phi_1) \cdot e^{-t/\tau_1} + g(t) \quad (2)$$

The factors P1 and P2 correspond to the amplitudes of modes oscillating at frequency ω_1 and ω_2, respectively. At pump photon energies below the band gap the phonon lifetime is observed to be long compared to the delay range covered by our measurements, and therefore no damping factor is added in equation (1). While using equation (1) to fit the data measured from the flake, only one oscillation was observed and thus we set P2=0. When fitting the date for photon energies above the bandgap we also observed a single frequency component. Additionally, due to the reduction of the phonon lifetime, the oscillation is multiplied by an exponential decay, which explains the form of equation (2).

To model the incoherent background we used the formula:

$$g(t) = A_{exp}e^{-t/\tau} + A_{lin}t + c \qquad (3).$$

We thus assume the incoherent background to consist of an exponential plus a correction of typically first order. The correction term is necessary as the maximum delay in the pump-probe traces can be smaller than the characteristic time scales of the background signal. We expand the correction term to maximum the third order for the case of the long-timescale scans reported in Figure 4(b) of the main text and Figure S4. This procedure is justified by the fact that we are solely interested in evaluating the coherent contribution to the signal.

For all traces, we set the position of the zero time-delay (t = 0) to the minimum of the rotation signal at the pump-probe overlap. To extract the temperature and pump photon energy dependence of the amplitudes P1 and P2 and of the incoherent background parameter *c*, we determined the frequencies ω_1 and ω_2 from the measure shown in Figure S4(a) and left all remaining parameters free.

Figure S2 shows an exemplary fit of a measurement from the bulk sample at 10 K. The corresponding fit-parameters are listed in the table below (table S1):

Table S1. Fit parameters.

P1		Φ_1	P2	Φ_2
(0.097 ± 0.008) mdeg		(0.03±0.08) rad	(0.041±0.007) mdeg	(0.5±0.1)rad
A_{exp}	τ	A_{lin}	c	
(-1.4±0.6) mdeg	(45±1) fs	(8±6) ×10^{-3} mdeg/ps	(-2.56±0.01) mdeg	

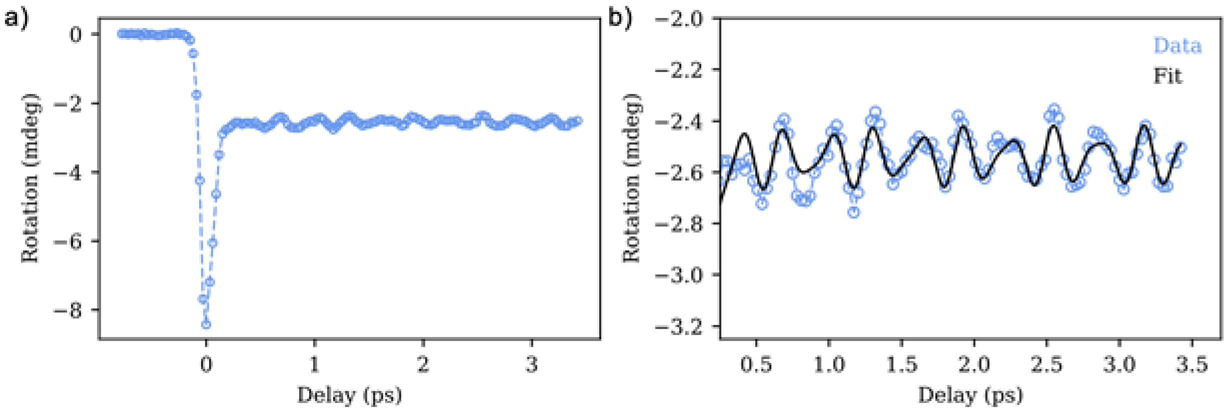

Figure S2. (a) Pump-probe trace at 10 K obtained employing a pump photon-energy of 1.03 eV. (b) Close-in view of the superimposed oscillations and the fit function from Eq. (1)

III. FOURIER SPECTRA

As an additional method to extract the frequency of the coherent oscillations in our measurements we isolated the oscillating signal by removing the incoherent background estimated using equation (3). We then performed a fast Fourier transformation on the remaining signal. Figure S3 presents an exemplarily Fourier transform analysis as performed on a pump probe-trace of the bulk crystal. Contributions at 3.2 THz and 4.8 THz in the Fourier spectra can be seen.

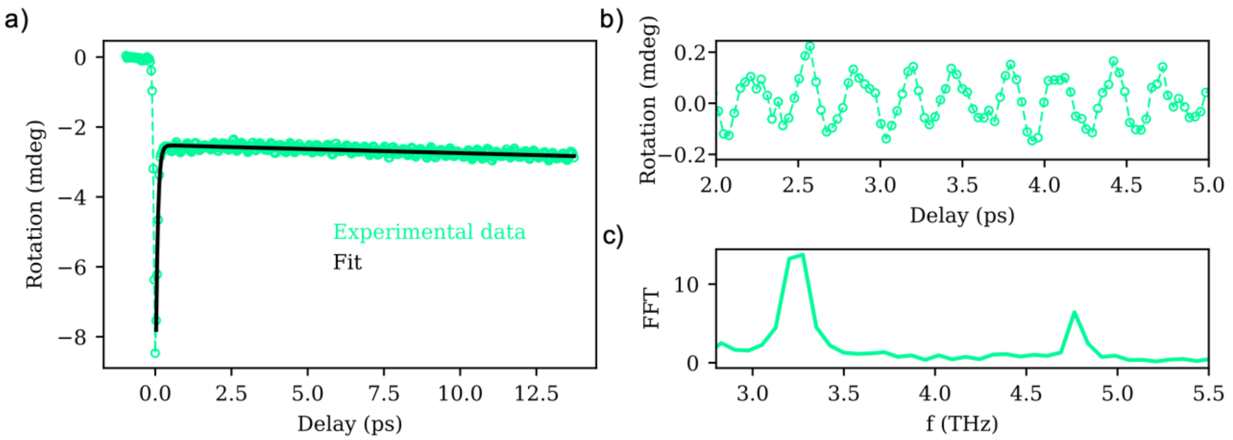

Figure S3. (a) Rotation of the polarization induced by a 1.03 eV pump beam at T=10 K. The best fit to the incoherent background, obtained with Eq. (3) is illustrated with the black line. (b) Phonon-induced oscillations isolated from the incoherent background. (c) Fast Fourier transformation of the trace shown in b).

IV. PHONON FREQUENCY AND LIFE-TIME

We extract the phonon lifetimes given in the main text from the data shown in Figure S4. The time-traces are measured over a long delay range (25 ps) in the bulk crystal at a temperature of T=10 K for pump-photon energies below (1.03 eV) and above (1.9 eV) the bandgap. After subtraction of the incoherent background, we fit the oscillations with two decaying sine waves for the excitation below the bandgap and with one sine wave for excitation above the band gap. This results in the lifetimes listed in table S2 below:

Table S2. Phonon lifetimes.

Photon Energy	Lifetime oscillation ω_1	Lifetime oscillation ω_2
1.03 eV	21 ps +-2 ps	12 +- 2 ps
1.9 eV	5 ps +- 1 ps	-

Thanks to the long delay range of these measurements, they can be used to exactly determine the frequency of the phonon modes. To this end, we kept the frequencies ω_1 and ω_2 as free parameter in the fit. We obtained:

ω_1 = (3.2407 ± 0.0006) THz and ω_2 = (4.780 ± 0.002) THz. In the main text, ω_1 is referred to as ω(0 T).

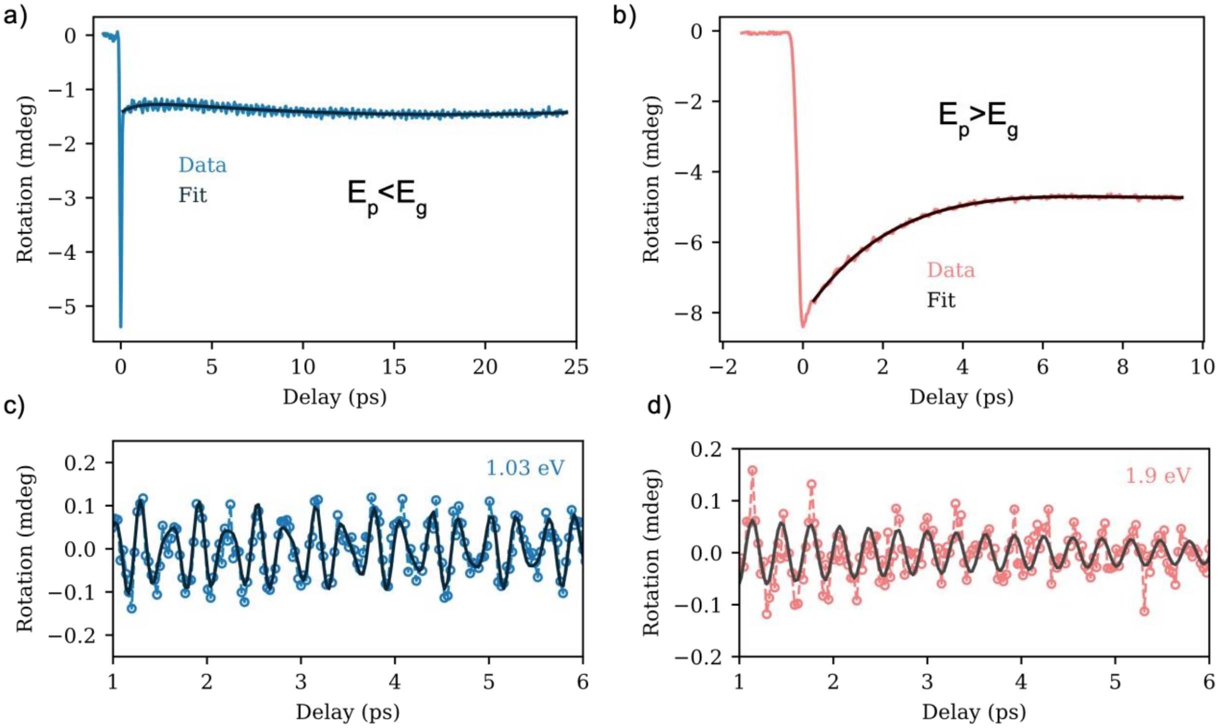

Figure S4. Extended pump-probe traces and the background fit (black curve) at pump photon energies of (a) 1.03 eV and (b) 1.9 eV. (c) Fit of the Phonon-induced oscillations to the signal without the background contribution at 1.03 eV and (d) 1.9 eV.

V. TRANSIENT REFLECTIVITY

Coherent oscillations have been also observed in the transient reflectivity signal shown in Figure S5 together with on exemplarily Fourier transform of the data recorded for the pump photon energy of 1.9eV.

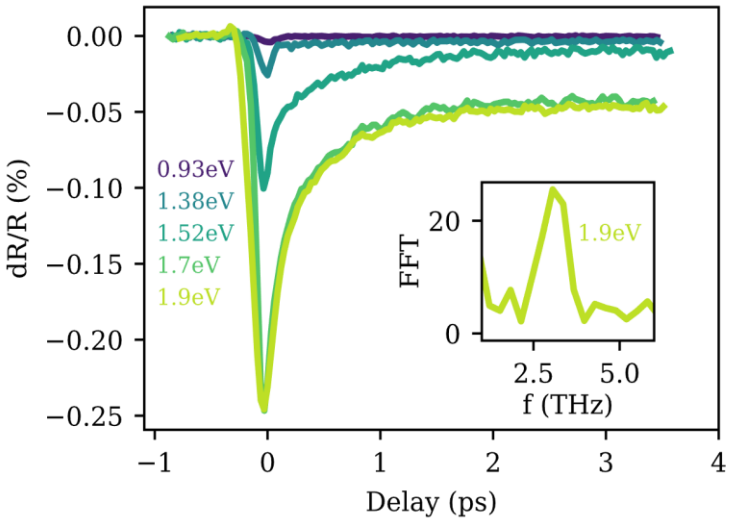

Figure S5 Transient reflectivity obtained employing different pump-photon energies in the bulk crystal at T=10 K. The inset shows the Fourier spectra for an exemplary trace.

In the Raman spectrum of $FePS_3$, further peaks between ~80 cm^{-1} (2.4 THz) and 160 cm^{-1} (4.8 THz) have been reported[1]. Although all Raman active modes can be in principle excited optically, not all of them can be simultaneously detected, since the rotation of the polarisation is sensitive only to some components of the dielectric and Raman tensor. In particular, the phonon modes at 3.2 THz and 4.8 THz consist mainly of in-plane oscillations of the Fe ions, modulating the magnetic zig-zag pattern and inducing birefringence or dichroism, thus directly affecting the polarisation state of light. The other Raman-active phonon modes, reported for example in Ref. [1], are dominated by out of plane displacements and do not affect the polarization of the probe beam.

VI. Pump-pulse polarization dependency

Figure S6 shows the dependence of the amplitude of the 3.2 THz and 4.8 THz modes in the bulk crystal at T=10 K from the (linear) polarization of the pump beam.

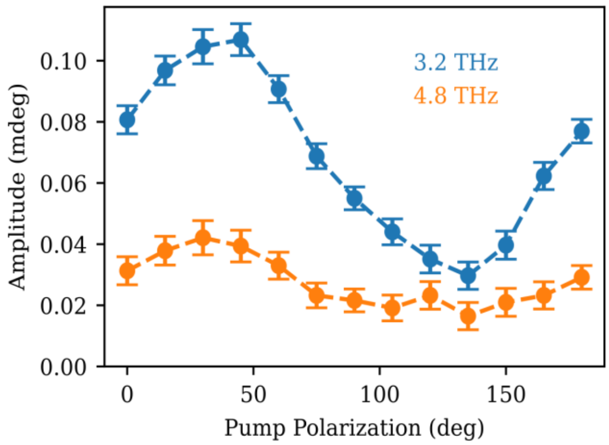

Figure S6. Pump-polarization dependence of the 3.2 THz and 4.8 THz phonon-amplitude in the bulk crystal at T = 10 K. The pump photon-energy was set to 1.03 eV while the probe was 1.45 eV.

VII. CRYSTAL GROWTH AND CHARACTERISATION

The crystal growth of $FePS_3$ was performed following a typical solid state reaction. Powders of Fe (99.998 %, from Alfa-Aesar), P (> 99.99 %, from Sigma-Aldrich) and S (99.998 %, from Sigma-Aldrich) were mixed in a stoichiometric ratio, sealed in an evacuated quartz ampoule (P ~$5 \cdot 10^{-5}$ mbar, length = 25 cm, internal diameter = 1.5 cm) and heated from room temperature to 400 °C at 1.1 °C/min. Then, the temperature was kept constant for twenty days and slowly cooled down (0.07 °C/min).

For obtaining large crystals, 4 mmol of the previous material was mixed with iodine as a transport agent ($[I_2]$ ~ 5 mg/cm^3) in an evacuated quartz ampoule (P ~ $5 \cdot 10^{-5}$ mbar, length = 50 cm, internal diameter = 1.4 cm). The quartz tube was placed inside a three-zone furnace with the material in the leftmost zone. The other two zones were heated up in 24 h from room temperature to 650 °C and kept for one day. After this, the leftmost side was heated up to 700 °C in 3 h and it was established in the three-zone furnace a gradient of 700 °C/650 °C/675 °C. Then the temperature was kept constant for 28 days and cooled down naturally. With this process we could obtain crystals with a length up to some

centimeters. The obtained crystals were analyzed by ICP-OES (Inductively Coupled Plasma - Optical Emission Spectrometry) and powder X-ray diffraction. The amount of elements obtained in weight are 30.0 ± 1.0 % for Fe, 15.7 ± 0.5 % for P and 51 ± 2 % for S, in good agreement with the expected ones (Fe: 30.5 %, P: 16.9 % and S: 52.6 %). The refinement (ICSD 61392) of the X-ray pattern revealed a monoclinic C face center crystal system with C12/m1 space group and a unit cell determined by $\alpha = \gamma = 90°$ and $\beta = 107.13(1)°$, a = 5.939(6) Å, b = 10.296(3) Å and c = 6.716(3) Å (Figure S7). The obtained results are in accordance with the ones reported in the literature.[2]

Magnetically, the crystals order antiferromagnetically at ca. 120 K (Figure S8), as verified by variable-temperature (2–300 K) direct current (d.c.) magnetic susceptibility measurements (carried out in an applied field of 1.0 kOe in a SQUID magnetometer from Quantum Design MPMS-XL-5), in accordance with previous reports.[3]

In order to verify the integrity of thin-layers, bulk $FePS_3$ was mechanically exfoliated, as typically performed in graphene and other two-dimensional materials.[4] The thin-layers were placed on top of 285 nm SiO_2/Si substrates and inspected by optical microscopy (Nikon Eclipse LV-100), atomic force microscopy (Nanoscope Iva Multimode Scanning Probe Microscope, Bruker, in tapping mode) and Raman spectroscopy (micro-Raman model XploRA ONE from Horiba, with a grating of 2400 gr/mm, slit of 50 μm, and hole of 500 μm and incident wavelength of 532 nm) (Figure S9). Six clear Raman modes are resolved, in agreement with the reported values in the literature.[1,5–7] The magnetic ordering of exfoliated thin-layers was probed by employing nanomechanical resonators (as reported by some us in references [8] and [9]) as well as its photo-electrical behavior (see references [10,11]).

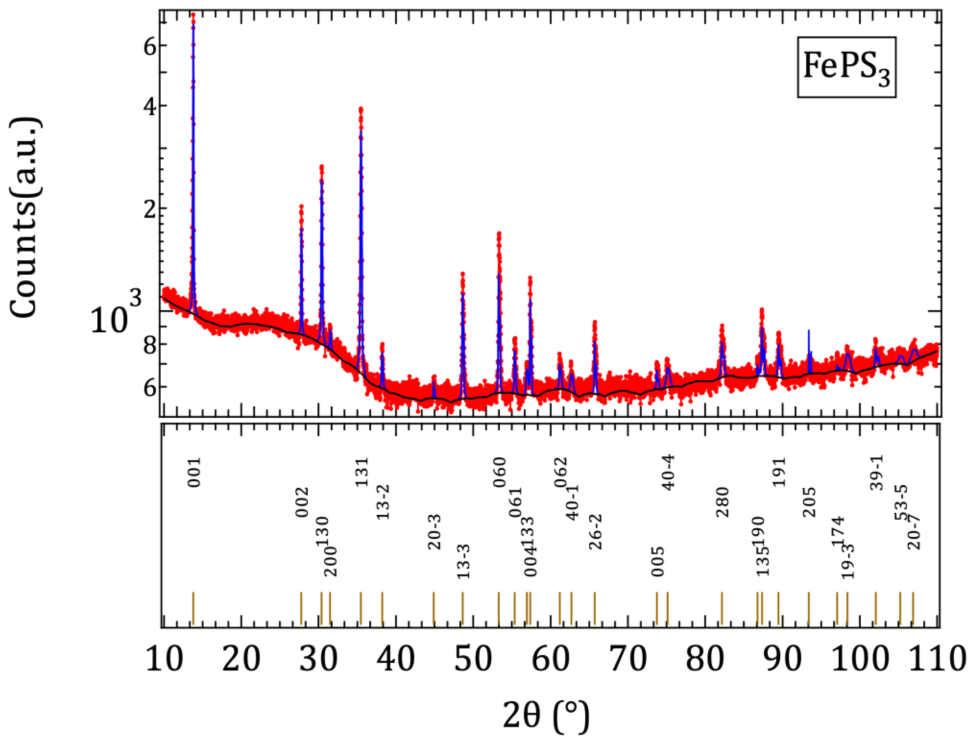

Figure S7. Experimental X-ray powder diffraction pattern (red) and corresponding fit (peaks in blue and background in black; χ2 = 1.5 × 10−6) for a grounded FePS3 crystal. Peak positions have been marked with brown lines.

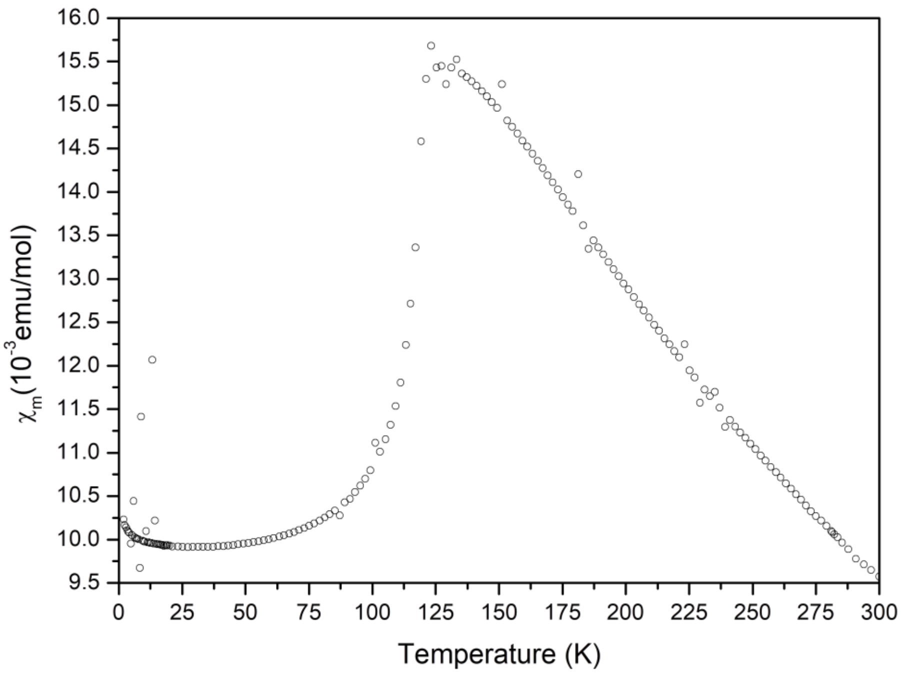

Figure S8. Thermal dependence of magnetic susceptibility in the temperature range 2–300 K.

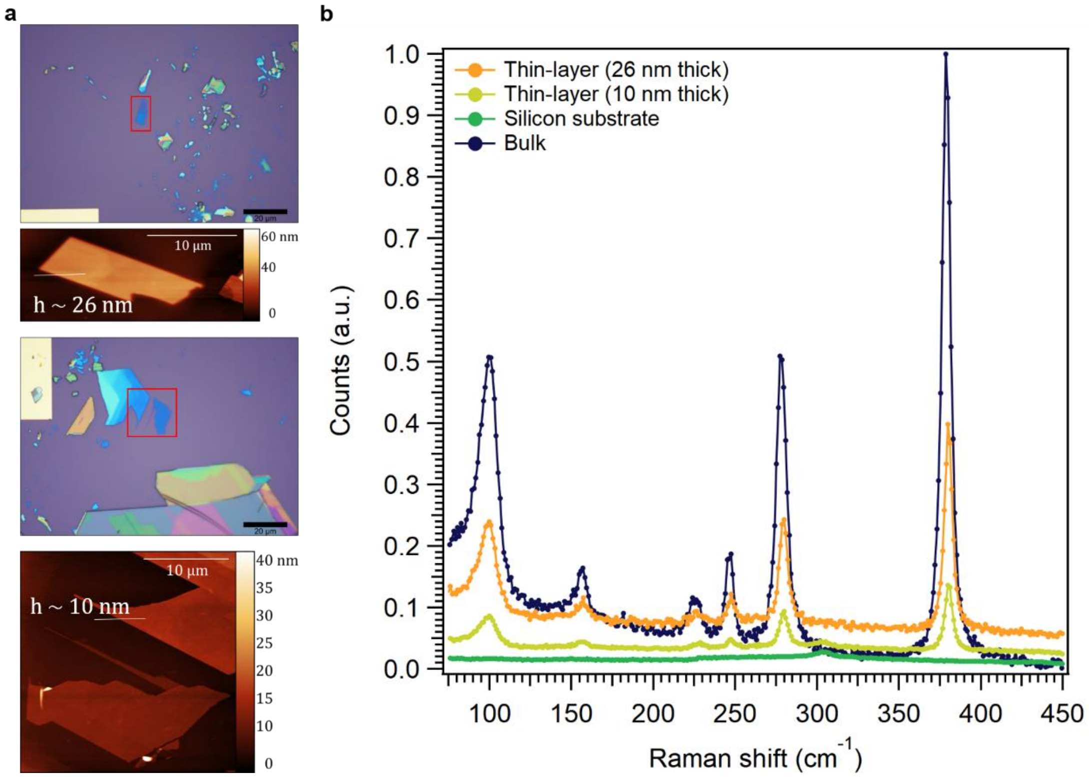

Figure S9. a) Optical microscopy (scale bar: 20 µm) of exfoliated FePS3 flakes together with atomic force microscopy images (scale bar: 10 µm) of the areas enclosed in red. b) Raman spectra of FePS3.

REFERENCES

1. Lee, J.-U. *et al.* Ising-Type Magnetic Ordering in Atomically Thin FePS3. *Nano Lett.* **16**, 7433–7438 (2016).
2. Ouvrard, G., Brec, R. & Rouxel, J. Structural determination of some MPS3 layered phases (M = Mn, Fe, Co, Ni and Cd). *Materials Research Bulletin* **20**, 1181–1189 (1985).
3. Brec, R. Review on structural and chemical properties of transition metal phosphorous trisulfides MPS3. *Solid State Ionics* **22**, 3–30 (1986).

4. Boix-Constant, C. *et al.* Out-of-Plane Transport of 1T-TaS 2 /Graphene-Based van der Waals Heterostructures. *ACS Nano* **15**, 11898–11907 (2021).

5. Wang, X. *et al.* Raman spectroscopy of atomically thin two-dimensional magnetic iron phosphorus trisulfide (FePS3) crystals. *2D Mater.* **3**, 031009 (2016).

6. McCreary, A. *et al.* Quasi-two-dimensional magnon identification in antiferromagnetic FePS3 via magneto-Raman spectroscopy. *Phys. Rev. B* **101**, 064416 (2020).

7. Scagliotti, M., Jouanne, M., Balkanski, M., Ouvrard, G. & Benedek, G. Raman scattering in antiferromagnetic FePS3 and FePSe3 crystals. *Physical Review B* **35**, 7097–7104 (1987).

8. Šiškins, M. *et al.* Magnetic and electronic phase transitions probed by nanomechanical resonators. *Nature Communications* **11**, 2698 (2020).

9. Šiškins, M. *et al.* Tunable Strong Coupling of Mechanical Resonance between Spatially Separated FePS 3 Nanodrums. *Nano Letters* **22**, 36–42 (2022).

10. Ramos, M. *et al.* Ultra-broad spectral photo-response in FePS3 air-stable devices. *npj 2D Materials and Applications* **5**, 19 (2021).

11. Ramos, M. *et al.* Photoluminescence Enhancement by Band Alignment Engineering in MoS2/FePS3 van der Waals Heterostructures. *ACS Appl. Mater. Interfaces* **14**, 33482–33490 (2022).
